# RNA Modifications and Epitranscriptomics

**DOI:** 10.1016/j.gpb.2023.10.002

**Published:** 2023-10-19

**Authors:** Chengqi Yi, Jianhua Yang

**Affiliations:** 1 State Key Laboratory of Protein and Plant Gene Research, School of Life Sciences, Peking University, Beijing 100871, China; 2 Peking-Tsinghua Center for Life Sciences, Peking University, Beijing 100871, China; 3 Department of Chemical Biology and Synthetic and Functional Biomolecules Center, College of Chemistry and Molecular Engineering, Peking University, Beijing 100871, China; 4 MOE Key Laboratory of Gene Function and Regulation, State Key Laboratory of Biocontrol, School of Life Sciences, Sun Yat-sen University, Guangzhou 510275, China; 5 The Fifth Affiliated Hospital, Sun Yat-sen University, Zhuhai 519000, China

More than 170 distinct chemical modifications have been identified in non-coding and coding RNAs. Accumulating evidence suggests that RNA modifications play pivotal roles at both the molecular and physiological levels. Dysregulation of RNA-modifying enzymes has been linked to various human cancers and developmental diseases. The expanding understanding of RNA modifications in molecular and cellular functions further suggests promising prospects for therapeutic applications. Recently, the creation of effective mRNA vaccines against coronavirus disease 2019 (COVID-19), based on RNA base modification, was honored with the Nobel Prize in Physiology or Medicine 2023 (https://www.nobelprize.org/prizes/medicine/2023/press-release/). Aiming to provide a forum for emerging advances in detection and functional studies of epitranscriptomic modifications, we have organized a special issue “RNA Modifications and Epitranscriptomics” for the journal *Genomics, Proteomics & Bioinformatics* (GPB). This special issue encompasses a wide range of topics, including: (1) dynamic landscapes of RNA modifications in various organisms, including animals, plants, and viruses; (2) mechanistic regulation of m^6^A and m^5^C modifications in human diseases and plant responses to stresses; (3) an online platform for unveiling the context-specific m^6^A methylation and m^6^A-affecting mutation; and (4) the regulatory role of non-coding RNAs (ncRNAs), including tRNAs and circular RNAs (circRNAs), in gene expression regulation.

We are pleased to present 14 articles selected for publication in this special issue, comprising eleven original research articles, one review, one letter, and one database article. An overview of the studies included in this issue is provided as follows.

Yafen Wang and Xiang Zhou reviewed the writer, reader, and eraser proteins involved in m^6^A modification, describing their mechanism of action during viral replication and infection. Moreover, the authors provided an overview of current detection methods for m^6^A, shedding light on the development of vaccines and antiviral drugs by examining the role of epigenetic modifications in viral processes [1].

Yixian Cun et al. uncovered a novel role of serine/arginine-rich splicing factor 7 (SRSF7) in regulating m^6^A and its impact on glioblastoma (GBM) progression. The authors found that SRSF7 specifically influenced m^6^A levels on genes associated with cell proliferation and migration, exhibiting oncogenic roles by recruiting the m^6^A methyltransferase complexes. This study highlights the significance of RNA-binding protein (RBP)-mediated specific regulation of m^6^A in determining cellular functions [2].

Xiao Han et al. presented the inaugural landscapes of dynamic DNA 5hmC and RNA m^5^C modifications across a variety of samples, including heart, kidney, liver, and lung, from human fetuses at 13–28 weeks. The authors identified 70,091 and 503 organ- and stage-specific differentially hydroxymethylated regions (DhMRs) and m^5^C-modified mRNAs, respectively. The integrated studies revealed a potential link between DNA modification and RNA methylation, illustrating the epigenetic dynamics during human fetal organogenesis [3].

Feng Yu et al. presented a study on the complexity of epitranscriptomic dynamics in rice by identifying RNA modifications using direct RNA sequencing (DRS) technology. Besides identifying tissue-specific genes and transcript expression, the authors also mapped the m^6^A and m^5^C modifications on RNA across six developmental tissues of rice, offering a thorough understanding of the rice transcriptome and epitranscriptome [4].

Peng Yu et al. explored the relationship between tRNA abundance and translational efficiency in mammals, as well as the contribution of tRNA expression to tissue-specific proteomes. The authors measured tRNA expression using demethylase-tRNA sequencing (DM-tRNA-seq) and mRNA translational efficiencies using ribosome-tagging sequencing (RiboTag-seq) in the mouse brain, heart, and testis. They showed tRNA expression variations among tissues and provided insights into the dynamics of tRNAs and their roles in translational regulation [5].

Shuai Chen et al. studied the presence and role of circRNAs in stress granules (SGs), which are cytoplasmic ribonucleoprotein assemblies formed under stress conditions. The authors used improved total RNA sequencing to identify both linear and circular RNAs in purified SG cores and found that circRNAs with higher SG-related RBP binding abilities are more likely to be enriched in SGs. They also identified differentially expressed SG-enriched circRNAs in hepatocellular carcinoma (HCC) and adjacent tissues, suggesting a regulatory role of circRNAs and SGs in HCC [6].

Bowen Song et al. presented m6A-TSHub, a comprehensive online platform designed to explore tissue-specific m^6^A RNA methylation patterns and related genetic mutations. This platform encompasses four core tools: m6A-TSDB, which curates extensive m^6^A site data from human tissues and tumors; m6A-TSFinder, a predictive server for tissue-specific m^6^A sites using deep learning; m6A-TSVar, evaluating genetic variant impacts on m^6^A modifications; and m6A-CAVar, cataloging mutations affecting m^6^A in various cancers. This serves as a pivotal resource for specialized m^6^A epitranscriptome studies [7].

Zidong Liu et al. uncovered the m^6^A modification dynamics during porcine spermatogenesis. Analyzing m^6^A distribution across spermatogonia, spermatocytes, and round spermatids, they identified a globally conserved m^6^A pattern in genes related to spermatogenesis. Enrichment of m^6^A in genes encoding metabolic enzymes and regulators was observed, showcasing its regulatory role. This study provides novel insights into the transcriptional regulation of lifelong male fertility in non-rodent mammals, enhancing our understanding of spermatogenesis in large animals [8].

Lorane Le Franc et al. employed the methylated RNA immunoprecipitation sequencing (MeRIP-seq) method to map m^6^A RNA methylomes during oyster development. Their analysis revealed dynamic and stage-specific m^6^A modifications in mRNA and lncRNA classes, displaying unique methylation patterns compared to transposon transcripts. The observed shifts in methylation profiles corresponded to expression changes across developmental stages such as cleavage, gastrulation, and organogenesis. These findings highlight the potential regulatory role of m^6^A in oyster development, offering novel insights into the control and evolution of developmental processes in lophotrochozoan organisms [9].

Ying Lv et al. deciphered the pattern and function of m^6^A modification during sexual reproduction in *Chlamydomonas* and also revealed its frequent occurrence in the DRAC motif and its main enrichment in the 3′ untranslated region (UTR) of mRNAs. The study found that m^6^A levels negatively correlate with gene expression, particularly affecting the microtubule-associated pathway. This study offers evolutionary insights into the role of m^6^A in *Chlamydomonas* and sheds light on its evolutionary significance in plant sexual reproduction [10].

Dan Song et al. highlighted that HCC tissues exhibit increased m^5^C methylation, particularly influencing phosphokinase signaling pathways. NOP2/Sun RNA methyltransferase (NSUN2) is notably overexpressed in HCC, impacting the expression of several genes and HCC cell sensitivity to the drug sorafenib. The study revealed innovative insights into the impact of RNA epigenetic modification on HCC progression, which might help to discover more effective HCC treatment targets and strategies [11].

Boyang Shi et al. employed the psoralen analysis of RNA interactions and structures method (PARIS) to map RNA structures in non-small cell lung cancer (NSCLC) cells, shedding light on epidermal growth factor receptor-tyrosine kinase inhibitor (EGFR-TKI) resistance mechanisms. They found that RNA structures, particularly in UTRs, correlate with translation efficiency, and the RNA structure of the gene encoding yrdC *N*^6^-threonylcarbamoyltransferase domain containing (YDRC) impacts EGFR-TKI sensitivity by modulating its translation. Disrupting the RNA structure in *YRDC* 3′ UTR with antisense oligonucleotide (ASO) presents a potential therapeutic strategy. This unveils a novel RNA structure-driven mechanism controlling EGFR-TKI resistance, providing valuable therapeutic perspectives [12].

Chen Zhu et al. explored the m^6^A-mediated regulatory impact on tea flavor-related metabolic pathways during solar-withering processes. Through integrated transcriptome analysis, the study revealed that two m^6^A erasers control global m^6^A levels, influencing terpenoid biosynthesis and spliceosome pathways. This m^6^A-mediated mechanism affects volatile terpenoid accumulation and flavonoid content. This study uncovered a novel epitranscriptomic layer in tea flavor formation, enhancing our understanding of tea flavor evolution during solar-withering [13].

Yongsheng Wang et al. systematically explored circRNAs in moso bamboo seedlings, especially in relation to gibberellin (GA) and auxin (NAA) treatments. They also developed a custom degradome sequencing method to detect microRNA-mediated cleavage of circRNAs. Their study revealed insights into the biogenesis, function, and microRNA-mediated degradation of circRNAs, emphasizing their significance in regulating hormone metabolism. The findings provided a deeper understanding of the role of circRNAs in plant biology, especially in moso bamboo [14].

These 14 articles in this special issue collectively broaden our understanding of RNA epitranscriptomics. They elucidate the roles of RNA modifications in gene regulation, diseases, and developmental processes across a range of organisms and tissues. We envision that new breakthrough in epitranscriptomics will underscore the complexity and importance of RNA modifications in determining cellular functions and hint at potential novel clinical applications.

## Completing interests

Both authors have declared no competing interests.

## CRediT authorship contribution statement

**Chengqi Yi:** Conceptualization, Writing – original draft, Writing – review & editing. **Jianhua Yang:** Conceptualization, Writing – original draft, Writing – review & editing. Both authors have read and approved the final manuscript.

## References

[1] Wang Y., Zhou X. (2023). *N*^6^-methyladenosine and its implications in viruses. Genomics Proteomics Bioinformatics.

[2] Cun Y., An S., Zheng H., Lan J., Chen W., Luo W. (2023). Specific regulation of m^6^A by SRSF7 promotes the progression of glioblastoma. Genomics Proteomics Bioinformatics.

[3] Han X., Guo J., Wang M., Zhang N., Ren J., Yang Y. (2023). Dynamic DNA 5-hydroxylmethylcytosine and RNA 5-methycytosine reprogramming during early human development. Genomics Proteomics Bioinformatics.

[4] Yu F., Qi H., Gao L., Luo S., Damaris R.N., Ke Y. (2023). Identifying RNA modifications by direct RNA sequencing reveals complexity of epitranscriptomic dynamics in rice. Genomics Proteomics Bioinformatics.

[5] Yu P., Zhou S., Gao Y., Liang Y., Guo W., Wang D.O. (2023). Dynamic landscapes of tRNA transcriptomes and translatomes in diverse mouse tissues. Genomics Proteomics Bioinformatics.

[6] Chen S., Zhang J., Zhao F. (2023). Screening linear and circular RNA transcripts from stress granules. Genomics Proteomics Bioinformatics.

[7] Song B., Huang D., Zhang Y., Wei Z., Su S., de Magalhães J.P. (2023). m6A-TSHub: unveiling the context-specific m^6^A methylation and m^6^A-affecting mutations in 23 human tissues. Genomics Proteomics Bioinformatics.

[8] Liu Z., Chen X., Zhang P., Li F., Zhang L., Li X. (2023). Transcriptome-wide dynamics of m^6^A mRNA methylation during porcine spermatogenesis. Genomics Proteomics Bioinformatics.

[9] Le Franc L., Petton B., Favrel P., Riviere G. (2023). m^6^A profile dynamics indicates regulation of oyster development by m^6^A-RNA epitranscriptomes. Genomics Proteomics Bioinformatics.

[10] Lv Y., Han F., Liu M., Zhang T., Cui G., Wang J. (2023). Characteristics of *N*^6^-methyladenosine modification during sexual reproduction of *Chlamydomonas reinhardtii*. Genomics Proteomics Bioinformatics.

[11] Song D., An K., Zhai W., Feng L., Xu Y., Sun R. (2023). NSUN2-mediated mRNA m^5^C modification regulates the progression of hepatocellular carcinoma. Genomics Proteomics Bioinformatics.

[12] Shi B., An K., Wang Y., Fei Y., Guo C., Zhang Q.C. (2023). RNA structural dynamics modulate EGFR-TKI resistance through controling *YRDC* translation in NSCLC cells. Genomics Proteomics Bioinformatics.

[13] Zhu C., Zhang S., Zhou C., Tian C., Shi B., Xu K. (2023). RNA methylome reveals the m^6^A-mediated regulation of flavor metabolites in tea leaves under solar-withering. Genomics Proteomics Bioinformatics.

[14] Wang Y., Wang H., Wang H., Zhou R., Wu J., Zhang Z. (2023). Multi-omics of circular RNAs and their responses to hormones in moso bamboo (*Phyllostachys edulis*). Genomics Proteomics Bioinformatics.

